# Multimodal analgesia with ropivacaine wound infiltration and intravenous flurbiprofen axetil provides enhanced analgesic effects after radical thyroidectomy: a randomized controlled trial

**DOI:** 10.1186/s12871-019-0835-2

**Published:** 2019-08-31

**Authors:** Xiaoxi Li, Ling Yu, Jiaonan Yang, Hongyu Tan

**Affiliations:** Key Laboratory of Carcinogenesis and Translational Research (Ministry of Education/Beijing), Department of Anesthesiology, Peking University Cancer Hospital & Institute, #52 Fucheng Street, Haidian District, Beijing, 100142 China

**Keywords:** Multimodal analgesia, Wound infiltration, Ropivacaine; flurbiprofen axetil, Postoperative analgesia, Thyroidectomy

## Abstract

**Background:**

Thyroidectomy is a common procedure that causes mild trauma. Nevertheless, postoperative pain remains a major challenge in patient care. Multimodal analgesia comprising a combination of analgesics and analgesic techniques has become increasingly popular for the control of postoperative pain. The present study tested the hypothesis that multimodal analgesia with combined ropivacaine wound infiltration and intravenous flurbiprofen axetil after radical thyroidectomy provided better analgesia than a single dosage of tramadol.

**Methods:**

This randomized controlled trial was conducted in a tertiary hospital. Forty-four patients (age, 18–75 years; American Society of Anesthesiologists status I or II; BMI < 32 kg/m^2^) scheduled for radical thyroidectomy were randomly assigned to a multimodal analgesia group (Group M) or a control group (Group C) by random numbers assignments, and 40 patients completed the study. All participants and the nurse in charge of follow-up observations were blinded to group assignment. Anesthesia was induced with sufentanil, propofol, and cisatracurium. After tracheal intubation, Group M received pre-incision wound infiltration with 5 ml of 0.5% ropivacaine mixed with epinephrine at 1:200,000 (5 μg/ml); Group C received no wound infiltration. Anesthesia was maintained with target-controlled infusion of propofol, remifentanil, sevoflurane, and intermittent cisatracurium. Twenty minutes before the end of surgery, Group M received 100 mg flurbiprofen axetil while Group C received 100 mg tramadol. Postoperative pain was evaluated with the numerical rating scale (NRS) pain score. Remifentanil consumption, heart rate, and noninvasive blood pressure were recorded intraoperatively. Adverse events were documented. The primary outcome was analgesic effect according to NRS scores.

**Results:**

NRS scores at rest were significantly lower in Group M than in Group C before discharge from the postoperative anesthetic care unit (*P* = 0.003) and at 2 (*P* = 0.008), 4 (*P* = 0.020), and 8 h (*P* = 0.016) postoperatively. Group M also had significantly lower NRS scores during coughing/swallowing at 5 min after extubation (*P* = 0.017), before discharge from the postoperative anesthetic care unit (*P* = 0.001), and at 2 (*P* = 0.002) and 4 h (*P* = 0.013) postoperatively. Compared with Group C, NRS scores were significantly lower throughout the first 24 h postoperatively in Group M at rest (*P* = 0.008) and during coughing/swallowing (*P* = 0.003). No serious adverse events were observed in either group.

**Conclusion:**

Multimodal analgesia with ropivacaine wound infiltration and intravenous flurbiprofen axetil provided better analgesia than tramadol after radical thyroidectomy.

**Trial registration:**

Chinese Clinical Trial Registry (registration number # ChiCTR1800020290; date of registration: 22/12/2018).

## Background

Thyroidectomy is a common procedure that causes mild trauma. Nevertheless, postoperative pain remains a major challenge in patient care. Optimizing postoperative pain management is an important goal in the perioperative period. Tramadol is a synthetic opioid frequently used to treat moderate pain. In contrast to pure opioid agonists, tramadol has a low risk of respiratory depression and sedative effects [1–4]. At our institution, tramadol is commonly prescribed to provide postoperative analgesia in patients undergoing thyroid surgery because it is effective in relieving mild to moderate pain, causes less respiratory depression and sedation than other opioids, and has a relatively low cost. However, tramadol has major adverse effects, including dizziness, nausea, and vomiting, which affect its clinical application and patient satisfaction [5]. Clinical research shows that postoperative nausea and vomiting (PONV) is primarily caused by the use of inhalational anesthesia and opioid analgesics [6]. Studies have shown that the incidence of PONV is reduced by the use of antiemetic drugs such as 5-HT3 receptor antagonists (e.g., dolasetron) and/or total intravenous anesthesia [7–9]. However, despite impressive advances in the field of anesthesia, PONV remains an unpleasant postoperative experience that must be considered.

Postoperative analgesia can be improved by combination therapies targeting different sites of the pain pathway. Moreover, multimodal analgesia can decrease opioid consumption and adverse effects. Therefore, multimodal analgesia using a combination of analgesics and analgesic techniques has become increasingly popular for the control of postoperative pain. Investigations on multimodal analgesia have been carried out in upper extremity surgery, hip and knee arthroplasty, cardiac surgery, and other major operations [10–14]. However, studies on multimodal analgesia in neck surgery remain limited.

Some inflammatory mediators released by damaged cells at the surgical site act directly on the nociceptor terminal to produce pain, while others lead to sensitization of the nociceptor terminal. Therefore, it has been proposed a multimodal analgesic regimen that includes anti-inflammatory drugs be used to control postoperative pain. Flurbiprofen axetil is a non-steroidal anti-inflammatory drug (NSAID) with a high affinity for inflamed tissues and a promising analgesic effect [15]. Local anesthetic wound infiltration has also been shown to reduce postoperative pain and opioid requirement in patients undergoing thyroid surgery [16, 17]. Ropivacaine is a popular long-acting local anesthetic that is widely used for local anesthesia due to its reduced toxic potential in comparison with other local anesthetic agents [18].

This prospective, randomized controlled trial aimed to evaluate the analgesic efficacy of multimodal analgesia with pre-incision ropivacaine wound infiltration and intravenous flurbiprofen axetil in patients undergoing radical thyroidectomy. The hypothesis was that multimodal analgesia benefits patients undergoing radical thyroidectomy by providing good analgesic effects with a low incidence of adverse effects. This is the first trial to compare a multimodal regimen consisting of pre-incision ropivacaine wound infiltration and intravenous flurbiprofen axetil versus a single dose of tramadol in patients undergoing thyroid surgery.

## Methods

### General information

This prospective, randomized controlled trial was designed in adherence to the CONSORT guidelines and was conducted in a tertiary hospital in Beijing, China. Ethics committee approval was obtained from the Institutional Review Board at Peking University Cancer Hospital (no. 2018YJZ74) and the study was registered on Chinese Clinical Trial Registry (no. ChiCTR1800020290). All participants provided written informed consent. Patients scheduled for elective radical thyroidectomy with an American Society of Anesthesiologists grade of I or II, aged 18–75 years, and a BMI < 32 kg/m^2^ were enrolled. Random numbers generated by Statistical Package for Social Sciences version 17.0 (SPSS Inc., Chicago, IL, USA) were used to randomly assign patients to the multimodal analgesia group (Group M) or the control group (Group C) in a 1:1 ratio. Patients were blinded to group assignment. A research nurse placed the random numbers in sealed envelopes. A resident who was independent of the recruitment process opened each patient’s envelope after all baseline assessments had been completed. Patients with the following conditions were excluded from the study: 1) history of chronic pain or chronic use of analgesics; 2) intake of NSAIDs, opioids, or other analgesics in the 24 h before surgery; 3) history of allergic reaction to NSAIDs; 4) any contraindications to flurbiprofen axetil, such as coagulation disorders, gastrointestinal ulceration, severe hypertension, severe cardiovascular or cerebrovascular disease, or renal dysfunction; 5) pregnancy or lactation; 6) inability to comprehend the concept of the numeric rating scale (NRS; 0, no pain; 10, worst pain imaginable); 7) lateral neck dissection during surgery; and 8) refusal to participate in the study.

### Anesthesia

Patients were placed in supine position on the operating table, with the neck hyperextended. Standard monitoring, including electrocardiography, heart rate (HR), noninvasive blood pressure (NBP), pulse oximetry, and bispectral index to monitor depth of anesthesia, were established before anesthetic induction. Anesthesia was induced with intravenous sufentanil (0.3 μg/kg), propofol (2–2.5 mg/kg), and cisatracurium (0.2 mg/kg). Tracheal intubation was performed after sufficient muscle relaxation was achieved. Patients were mechanically ventilated to maintain end-tidal carbon dioxide between 35 and 45 mmHg. After tracheal intubation, patients in Group M received subcuticular wound infiltration with 5 ml of 0.5% ropivacaine mixed with epinephrine at a ratio of 1:200,000 (5 μg/ml) prior to skin incision. Group C received no wound infiltration. General anesthesia was maintained with target-controlled infusion (Graseby 3500; AstraZeneca, UK) of propofol (2.0 μg/ml, plasma concentration), remifentanil (3.0–4.0 ng/ml, plasma concentration), and sevoflurane (1.0–2.0%, end-tidal concentration) to maintain a spectral entropy value of 40 to 60. Muscle relaxation was achieved with intermittent cisatracurium. All patients received lactated Ringer’s solution. If blood pressure fell to 30% below baseline for more than 1 min, fluid infusion was accelerated or 6 mg ephedrine was administered. Surgeries were performed by the same surgical team with the same standardized technique. At 20 min before the end of surgery, Group M received 100 mg flurbiprofen axetil (Beijing Taide Pharmaceutical Co., Ltd.) while Group C received 100 mg tramadol intravenously (slowly injected during a 5-min period), followed by injection of 12.5 mg dolasetron in both groups to prevent PONV. Sevoflurane was discontinued 15 min before the completion of surgery. Propofol/remifentanil infusion was terminated at the end of surgery. Muscle relaxation was antagonized with 1 mg intravenous atropine and 2 mg neostigmine. Patients were extubated after responding to verbal commands and achieving adequate spontaneous ventilation. Patients were then transferred to the postoperative anesthetic care unit (PACU) for further observation until they fulfilled discharge criteria.

### Measurements

Hemodynamic parameters, including HR and NBP, were documented at specific timepoints: before induction (T1), 3 min after tracheal intubation (T2), at the beginning of surgery (T3), after 10 min of surgery (T4), after 30 min of surgery (T5), at the end of surgery (T6), immediately after extubation (T7), and before discharge from the PACU (T8).

Acute postoperative pain was assessed in accordance with the NRS score under two conditions (at rest and during coughing/swallowing) at 5 min after tracheal extubation, before patient discharge from the PACU, and at 2, 4, 8, 12, 24, and 48 h after surgery. If a patient had moderate pain (NRS score of 4–6), 50 mg flurbiprofen axetil was prescribed. If a patient had severe pain (NRS score above 6), 100 mg tramadol was administered as a rescue analgesic. All adverse events related to the administered agents, such as dizziness and PONV, were documented. If a patient experienced severe nausea or vomiting, metoclopramide was administered as a rescue antiemetic agent. Follow-up observations were performed by a nurse from the PACU who was not involved in the study and who was blinded to group assignment.

### Outcomes

The primary outcome was analgesic effect in accordance with the NRS scores at specific timepoints. The secondary outcomes were intraoperative remifentanil consumption, the need for postoperative rescue analgesia, adverse effects, and hemodynamic response during surgery.

### Sample size

A preliminary trial conducted by the authors found that the average NRS score within 48 h after surgery was 0.92 ± 0.53 in Group M and 1.48 ± 0.62 in Group C. With this information, a sample size of 15 patients per group was estimated to have at least 80.0% power at a significance level of 5%, according to Power Analysis and Sample Size software (version 11.0; NCSS, LLC, Kaysville, UT, USA).

### Statistical analysis

SPSS was used for statistical analysis. Numerical variables are shown as mean ± standard deviation (SD) or median, in accordance with their distribution. Categorical variables were analyzed with the Pearson chi-squared test. Continuous variables were analyzed with independent-samples t-test or the rank sum test, in accordance with their distribution. Hemodynamic responses at different timepoints were compared with repeated-measures analysis of variance. A value of *P* < 0.05 was considered statistically significant.

## Results

**Fig. 1:**
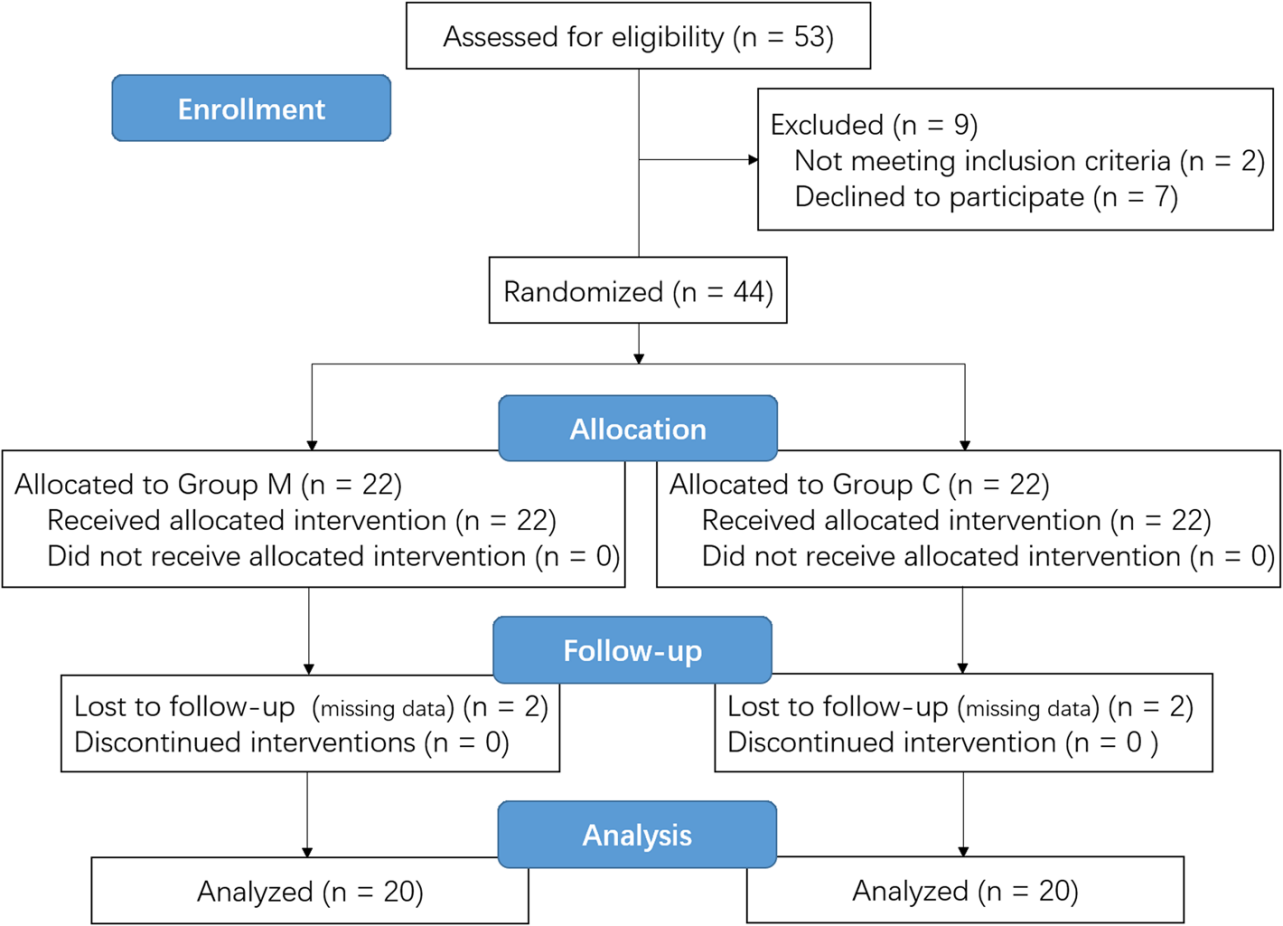
Flow diagram

Forty-four patients were randomized from January to April 2019 (Fig. 1). Four patients were excluded due to missing data. Therefore, 40 patients were analyzed with 20 patients in each group. There were no significant differences between the groups in demographic characteristics or intraoperative data (Table 1). Compared with Group C, Group M had a lower mean blood pressure (MBP; *P* = 0.019) (Table 2). However, no significant differences were found between groups in HR (*P* = 0.119) or the use of vasoactive drugs (*P* = 0.507). In addition, the two groups did not differ in infusion volume (*P* = 0.634) or blood loss (*P* = 0.515).

**Table 1:** Demographic characteristics and intraoperative data

	Group M (n = 20)	Group C (*n* = 20)	Statistics	*P*
Age (years)	42.4 ± 8.7	39.5 ± 8.4	1.066^a^	0.293
Sex (M/F)	4/16	6/14	0.533^b^	0.465
Weight (kg)	65.4 ± 10.3	63.6 ± 12.4	0.479^a^	0.635
BMI (kg/m^2^)	24.2 ± 3.9	23.3 ± 3.6	0.801^a^	0.428
Type of operation, n (%)				
Hemithyroidectomy	13 (65.0%)	10 (50.0%)	0.921^b^	0.337
Total thyroidectomy	7 (35.0%)	10 (50.0%)		
Duration of surgery (s)	68.5 ± 32.2	66.7 ± 23.2	0.203^a^	0.840
Consciousness recovery time (s)	7.0 ± 4.1	6.4 ± 3.5	0.543^a^	0.590
Extubation time (s)	10.0 ± 5.5	11.1 ± 5.9	0.582^a^	0.564
Remifentanil (mg)	0.626 ± 0.268	0.645 ± 0.238	0.238^a^	0.813

Data are presented as mean ± SD or *n* (%). Group M, multimodal analgesia group who received ropivacaine locally plus intravenous flurbiprofen axetil; Group C, control group who received a single dose of tramadol; *BMI* Body mass index; ^a^, *t* value; ^b^, Chi-square value

**Table 2:** Mean blood pressure during surgery

	Group M (*n* = 20)	Group C (n = 20)	Statistic	*P*
Before induction (T1)	94.7 ± 13.5	95.0 ± 9.1	7.187	0.019*
3 min after induction (T2)	75.4 ± 15.3	74.7 ± 10.4		
At the beginning of surgery (T3)	66.9 ± 9.4	71.8 ± 7.0		
After 10 min of surgery (T4)	71.8 ± 5.8	78.6 ± 9.1		
After 30 min of surgery (T5)	69.1 ± 7.8	77.4 ± 10.6		
At the end of surgery (T6)	72.2 ± 11.5	83.8 ± 12.3		
Immediately after extubation (T7)	94.3 ± 11.7	99.2 ± 11.7		
Before discharge from the PACU (T8)	85.8 ± 8.1	91.5 ± 11.9		

Data are presented as mean ± SD. Group M, multimodal analgesia group who received ropivacaine locally plus intravenous flurbiprofen axetil; Group C, control group who received a single dose of tramadol; PACU, postoperative anesthetic care unit; *, *P* < 0.05

**Fig. 2:**
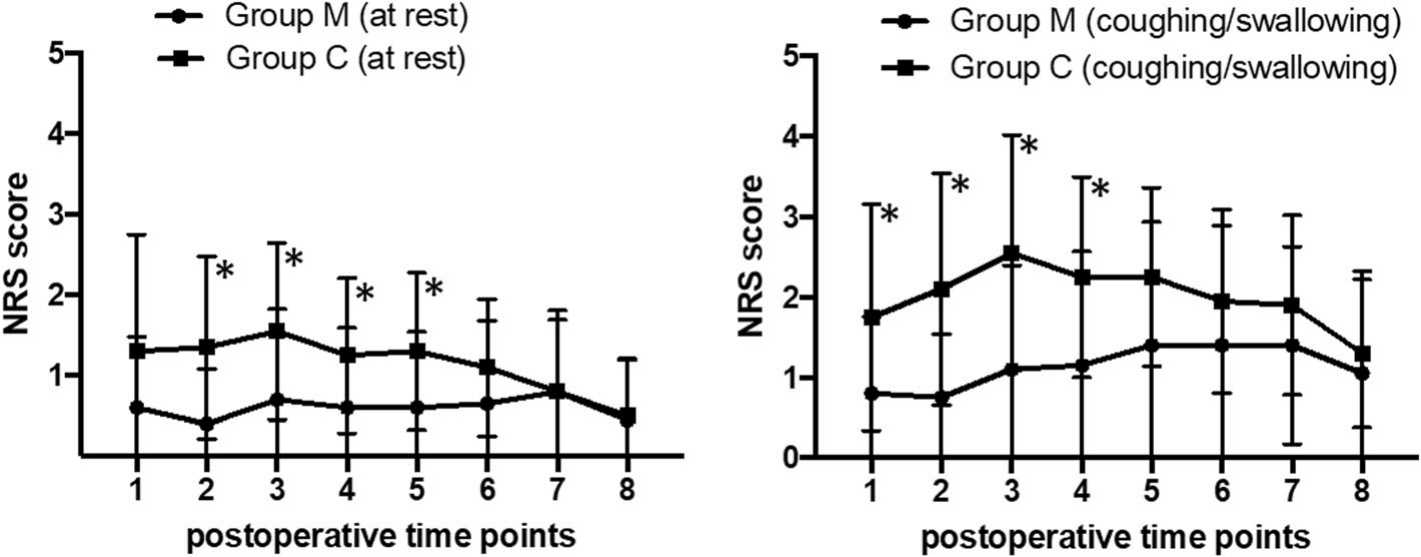
Numerical rating scale (NRS) pain scores at various postoperative timepoints. NRS scores at rest were significantly lower in Group M than Group C before patient discharge from the PACU (*P* = 0.003), and at 2 (*P* = 0.008), 4 (*P* = 0.020), and 8 h (*P* = 0.016) postoperatively. NRS scores during coughing/swallowing were significantly lower in Group M than Group C at 5 min after tracheal extubation (*P* = 0.017), before patient discharge from the PACU (*P* = 0.001), and at 2 (*P* = 0.002) and 4 h (*P* = 0.013) postoperatively. Group M, multimodal analgesia group who received ropivacaine locally plus intravenous flurbiprofen axetil; Group C, control group who received a single dose of tramadol; PACU, postoperative anesthetic care unit; *P < 0.05

Postoperative NRS scores are presented in Fig. 2 and Table 3. There was no significant difference between groups in the number of patients requiring additional analgesia postoperatively (*P* = 1.000).

**Table 3:** Average numerical rating scale (NRS) pain scores during the first 48 h postoperatively

	Group M (*n* = 20)	Group C (n = 20)	Statistics	*P*
During the first 24 h postoperatively				
At rest	0.614 ± 0.620	1.236 ± 0.777	2.795^a^	0.008*
During coughing or swallowing	1.143 ± 0.834	2.107 ± 1.057	3.203^a^	0.003*
During the first 24–48 h postoperatively				
At rest	0.000	0.500		0.662
During coughing or swallowing	1.225 ± 1.186	1.600 ± 0.968	1.095^a^	0.280
Average scores under both conditions within 48 h postoperatively	0.863 ± 0.647	1.575 ± 0.794	3.112^a^	0.004*

Data are presented as mean ± SD or median. Group M, multimodal analgesia group who received ropivacaine locally plus intravenous flurbiprofen axetil; Group C, control group who received a single dose of tramadol. ^a^, *t* value; *, *P* < 0.05

No serious adverse events related to the agents used in the present study were observed. The incidence of adverse effects within the 48 h postoperative period was not significantly different between the groups (Table 4).

**Table 4:** Incidence of adverse effects

	Group M (n = 20)	Group C (*n* = 20)	χ^2^	*P*
Dizziness	3 (15.0%)	3 (15.0%)	0.000	1.000
Nausea	4 (20.0%)	3 (15.0%)	0.000	1.000
Vomiting	1 (5.0%)	1 (5.0%)	0.000	1.000

Data are presented as *n* (%). Group M, multimodal analgesia group who received ropivacaine locally plus intravenous flurbiprofen axetil; Group C, control group who received a single dose of tramadol

## Discussion

Flurbiprofen axetil is an injectable non-selective cyclooxygenase inhibitor. It is a prodrug prepared by enveloping flurbiprofen ester in a drug carrier of lipid microspheres, which congregate selectively in inflammatory tissues with a high affinity and provide sustained drug release. A meta-analysis of randomized controlled trials showed that patients treated with preoperative flurbiprofen axetil had significantly lower postoperative pain scores than those who did not receive flurbiprofen axetil [15]. Regional techniques such as local anesthetic wound infiltration also reduce postoperative pain and opioid requirements [16, 17]. In thyroid surgery, local anesthetic wound infiltration is safe and easy to perform and has shown good analgesic effects in some studies [16, 19–22]; however, the results are controversial [23, 24]. In addition, the analgesic benefit of local wound infiltration seems to be maintained for only a short period of time after thyroid surgery [20, 25], and breast cancer surgery [26]; administration of local anesthetics significantly decreased pain only at 2 h postoperatively. Postoperative pain after total thyroidectomy reportedly reaches a maximum at 1 h postoperatively, and starts to decrease 3 h later [20]. Therefore, the first few hours following thyroidectomy are the most crucial for pain management.

To overcome the problems of the short duration of analgesia obtained with incisional infiltration and the insufficient analgesia provided by NSAIDs, we administered a combined analgesia protocol of ropivacaine wound infiltration plus intravenous flurbiprofen axetil. Ropivacaine was chosen for its longer block duration, lower toxicity, and greater safety compared with other local anesthetics; plasma levels and risks are associated with the total dose used and the extent of absorption [27]. According to previous studies, ropivacaine concentrations of 0.25, 0.5 and 0.75% provide adequate analgesia via wound infiltration [27–29]. Based on the literatures and our experiences, we chose to use 0.5% ropivacaine because it provides good analgesia with few adverse effects. To prolong local anesthetic action and reduce vascular absorption, epinephrine was added to the ropivacaine. However, epinephrine should be applied with caution in patients with severe hypertension, or cardiovascular and cerebrovascular diseases, as it may cause tachycardia and hypertension if absorbed intravascularly. In our study, postoperative pain reached its maximum at 2 h postoperatively in Group C, similarly to previous findings [20], and remained at a relatively high level until 8 h postoperatively. The pain scores of Group C at maximum (2 h postoperatively) were 1.6 at rest and 2.6 during coughing/swallowing. The average NRS pain scores of Group C were 1.4 at rest and 2.0 during coughing/swallowing during the 2–8 h postoperative period, whereas the average NRS pain scores of Group M were 0.6 at rest and 1.2 during coughing/swallowing. The postoperative pain score reached its maximum at 24 h postoperatively at rest and 8 h postoperatively during coughing/swallowing in Group M (0.6 at rest and 1.4 during coughing/swallowing). However, the maximum postoperative pain scores in Group M were still relatively low compared to that in Group C at the same observation timepoint. Thus, multimodal analgesia delayed the occurrence of maximum postoperative pain (the maximum pain score was still relatively low), and successfully achieved pain relief during the early postoperative period (2–8 h postoperatively). This is meaningful, as several studies have indicated that the first few hours following thyroidectomy are the most crucial for pain management because patients experienced maximum pain during this period. Our results also demonstrate that the analgesic effects in Group M were significantly better than those in Group C on the first postoperative day both at rest and during coughing/swallowing; the average NRS pain score in Group M was also significantly lower than that in Group C within the first 2 postoperative days. These results showed that multimodal analgesia enhanced postoperative pain relief not only during the early postoperative stage, but throughout the 48 h postoperative period. This is a meaningful improvement compared with several studies that reported insufficient maintenance of pain control after administration of local anesthesia [20, 25]. One of the adverse effects of tramadol is nausea, which may result in higher frequency of swallowing and could explain the higher NRS scores during coughing/swallowing in Group C versus Group M. However, the incidence of nausea did not significantly differ between the two groups, which means that the two groups had a similar frequency of swallowing. Therefore, we believe that the comparison between the two groups of pain scores during coughing/swallowing was not affected by nausea caused by tramadol.

Consistent with previous studies [30–33], 80.0% of patients had NRS scores lower than 4 (only one patient had a NRS score of greater than 6), indicating that thyroidectomy causes mild to moderate postoperative pain. Additional rescue analgesics were not usually required, and the need for additional rescue analgesics did not significantly differ between the groups. However, the intensity of postoperative pain may vary with surgical approach, anesthetic management, and pain-control protocols. In our study, the application of a small incision (approximately 4–7 cm) in thyroidectomy may have resulted in minimal pain in both groups. Our study showed that pain control after thyroid surgery can generally be accomplished with either a multimodal analgesia regimen of pre-incision wound infiltration and flurbiprofen axetil or with a single dose of tramadol, mostly without additional analgesics. Several previous studies suggested that local anesthetic wound infiltration decreases opioid consumption [16, 17, 34]. However, the present study found that intraoperative remifentanil use did not differ between the groups, consistent with a previous study [25].

Multimodal approaches to pain management have been shown to reduce adverse effects such as dizziness and PONV in patients undergoing surgical procedures [14, 35]. However, our results showed no significant differences in drug-associated adverse effects between the groups. In our study, the incidence of PONV was relatively low compared with data reported in a previous study [36]. PONV was only experienced by four (20.0%) patients in Group M and three (15.0%) in Group C. We assume that PONV was prevented by the combined use of dolasetron and propofol infusion (which allowed a lower concentration of sevoflurane during surgery). Furthermore, the relatively low incidence of PONV in Group C may be related to the slow injection of tramadol over a 5-min period. NSAIDs are associated with many adverse effects, including platelet aggregation inhibition, gastrointestinal mucosal injury, and renal failure. However, no adverse events were observed in our study. There were no significant differences between the two groups in intraoperative blood loss, and none of the patients experienced postoperative hemorrhage, probably because only a single dose of flurbiprofen axetil injection was administered. Similar data have been reported in other studies [37, 38]. Overall, our results demonstrated a low incidence of adverse effects due to multimodal analgesia with ropivacaine wound infiltration and intravenous flurbiprofen axetil (both administered within their recommended doses and volume) in patients undergoing thyroidectomy.

A previous study showed that patients treated with preoperative local infiltration exhibited lower MBP than other patients [25]. Similar changes were observed in our study, with a lower MBP in Group M than in Group C. In the previous study, the MBP reduction was explained as the result of preoperative local infiltration [25]. However, in our study, there were no significant differences between groups in vasoconstrictor requirement during surgery, indicating that the two groups had similar proportions of patients with hemodynamic changes within 30% of base line. Therefore, the differences in MBP may not be considered clinically significant.

The present study had some limitations. A sex ratio disparity existed among the participants, as 30 of the 40 patients analyzed were female. This distribution may be inevitable, as thyroid cancer is more common in women [39]. Nevertheless, these results need to be confirmed in a larger trial.

## Conclusion

Multimodal analgesia with ropivacaine wound infiltration and intravenous flurbiprofen axetil improves the quality of postoperative analgesia in patients undergoing radical thyroidectomy, and has few adverse effects. This approach has advantages over tramadol for patients undergoing radical thyroidectomy. We recommend that this multimodal regimen be used in the clinical setting as described.

## Data Availability

The datasets used and/or analysed during the current study are available from the corresponding author on reasonable request.

## Abbreviations

HR: Heart rate
MBP: Mean blood pressure
NBP: Noninvasive blood pressure
NRS: Numerical rating scale
NSAID: Non-steroidal anti-inflammatory drug
PACU: Postoperative anesthetic care unit
PONV: Postoperative nausea and vomiting
SD: Standard deviation
SPSS: Statistical Package for Social Sciences
