# Correction to: Multimodal analgesia with ropivacaine wound infiltration and intravenous flurbiprofen axetil provides enhanced analgesic effects after radical thyroidectomy: a randomized controlled trial

**DOI:** 10.1186/s12871-019-0871-y

**Published:** 2019-11-13

**Authors:** Xiaoxi Li, Ling Yu, Jiaonan Yang, Hongyu Tan

**Affiliations:** Key Laboratory of Carcinogenesis and Translational Research (Ministry of Education/Beijing), Department of Anesthesiology, Peking University Cancer Hospital & Institute, #52 Fucheng Street, Haidian District, Beijing, 100142 China

**Correction to: BMC Anesthesiol (2019) 19:167**

**https://doi.org/10.1186/s12871-019-0835-2**

Following publication of the original article [1], the authors reported that there has been a mistake regarding the unit of duration of surgery, consciousness recovery time, and extubation time in Table 1; the correct unit is minute. This is the correct information in Table 1:

Duration of surgery (min)

Consciousness recovery time (min)

Extubation time (min)

## References

[1] Li X (2019). Multimodal analgesia with ropivacaine wound infiltration and intravenous flurbiprofen axetil provides enhanced analgesic effects after radical thyroidectomy: a randomized controlled trial. BMC Anesthesiol.

